# The True Dislocation Incidence following Elective Total Hip Replacement in Sweden: How Does It Relate to the Revision Rate?

**DOI:** 10.3390/jcm13020598

**Published:** 2024-01-20

**Authors:** Peter H. J. Cnudde, Jonatan Nåtman, Ola Rolfson, Nils P. Hailer

**Affiliations:** 1 Swedish Arthroplasty Register, Registercentrum Västra Götaland, 413 45 Gothenburg, Sweden; jonatan.natman@gmail.com (J.N.); ola.rolfson@vgregion.se (O.R.); nils.hailer@surgsci.uu.se (N.P.H.); 2 School of Management, Swansea University, Bay Campus, Swansea SA1 8EN, UK; 3 Department of Orthopaedics, Hywel Dda University Healthboard, Prince Philip Hospital, Bryngwynmawr, Llanelli SA14 8QF, UK; 4 Department of Orthopaedics, Institute of Clinical Sciences, University of Gothenburg, Göteborgsvägen 37, 431 80 Mölndal, Sweden; 5 Orthopaedics, Department of Surgical Sciences, Uppsala University, Akademiska Sjukhuset, Ingång 61, 751 85 Uppsala, Sweden

**Keywords:** total hip arthroplasty (THA), dislocation, register, revision, linkage

## Abstract

(1) Background: The true dislocation incidence following THA is difficult to ascertain in population-based cohorts. In this study, we explored the cumulative dislocation incidence (CDI), the relationship between the incidence of dislocation and revision surgery, patient- and surgery-related factors in patients dislocating once or multiple times, and differences between patients being revised for dislocation or not. (2) Methods: We designed an observational longitudinal cohort study linking registers. All patients with a full dataset who underwent an elective unilateral THA between 1999 and 2014 were included. The CDI and the time from the index THA to the first dislocation or to revision were estimated using the Kaplan–Meier (KM) method, giving cumulative dislocation and revision incidences at different time points. (3) Results: 136,810 patients undergoing elective unilateral THA were available for the analysis. The 30-day CDI was estimated at 0.9% (0.9–1.0). The revision rate for dislocation throughout the study period remained much lower. A total of 51.2% (CI 49.6–52.8) suffered a further dislocation within 1 year. Only 10.9% of the patients with a dislocation within the first year postoperatively underwent a revision for dislocation. (4) Discussion: The CDI after elective THA was expectedly considerably higher than the revision incidence. Further studies investigating differences between single and multiple dislocators and the criteria by which patients are offered revision surgery following dislocation are urgently needed.

## 1. Introduction

### 1.1. Background

Dislocation is a dreaded complication for both patient and healthcare providers and is one of the leading reasons for reoperations and revisions [1,2]. The causes for dislocations are multifactorial, and patients who suffer from single or multiple dislocations are usually less satisfied with their surgical results, they need further contact with healthcare providers, and many may need further surgical interventions [3,4,5,6]. These surgical interventions are not without risk, are not always able to correct the issue of instability, and come at a huge cost for the patient and society [7,8,9,10,11,12,13]. Despite the fact that dislocation after total hip arthroplasty (THA) is one of the most investigated questions in THA research, only recently has there been interest in describing the cumulative incidence of dislocations after elective THA and the underlying risk factors using population-based cohorts [5,14]. Using national register revisions for all reasons, and specifically revisions for dislocation, have been well studied [15,16,17,18,19,20,21]. Many studies, and specifically register studies, have published the revision rate for dislocation as a proxy for dislocation; the true dislocation incidence, the reoccurrence of dislocation, and the association between dislocation and revisions are less well investigated [14].

Our study group has previously reported on dislocations following arthroplasty in acute hip fracture patients [22,23]. As dislocations are usually not reported to national arthroplasty registers, additional data from other sources are a requirement. Many arthroplasty register studies also lack important information on some important confounders, such as neurological disease or spinal problems. By combining data from the national arthroplasty registers with data from national patient registers, it was possible to adjust for these confounders and to study outcomes that are not routinely recorded within national registers [24].

### 1.2. Rationale

The dislocation incidence following elective THA is not well researched in large, population-based cohorts, and register research has utilized revision for dislocation or instability as a proxy. Dislocation following elective THA has an enormous impact for the patient, the surgical teams, and healthcare systems; we believed that the dislocation incidence in Sweden needed to be studied. The primary aim of this study was to describe the dislocation incidence for a cohort of Swedish patients who underwent elective THA [3,12,13] and how the cumulative dislocation incidence related to the revision incidence for dislocation and to the revision incidence for any reason. As a single dislocation can become a reoccurring problem [4,14], we also intended to study the proportion of patients having multiple dislocations, whether the groups of single vs. multiple dislocations differed, and how many dislocations did occur before revision surgery. Finally, differences between patient- and surgery-related characteristics in the revised versus non-revised group were explored. Specifically, the research project studied the following research questions:

a. What is the cumulative dislocation incidence after elective primary THA in Sweden, and how does this compare to the revision incidence for dislocation or for any reason?
b. Is there a difference in patient- and surgery-related characteristics between patients dislocating once vs. multiple times within 1 year of index surgery?
c. Is there a difference in patient- and surgery related characteristics between patients revised vs. not revised for dislocation?

## 2. Materials and Methods

This research project is a longitudinal observational cohort study using prospectively recorded data from the Swedish Arthroplasty Register (SAR), linked with data from the National Patient Register (NPR) [24]. All patients undergoing THA are entered in the SAR database and are followed up over time, with a record of further surgical interventions on the ipsilateral or contralateral side or death. Any contact with the secondary care system (as inpatients since 1987 and any contact with outpatient services, day case interventions, and mental health care since 2001) will provide an entry with diagnostic and procedural codes in the NPR. In order to avoid the problems with the undefined laterality of the dislocation within the NPR, all patients with bilateral hip arthroplasties were excluded (if they were bilaterally operated when entering the observation period) or censored at the time of the second procedure if this occurred during the observation period. The SAR has high completeness for primaries (98%) and revisions (94%) and had full national coverage (100%) in 2014 [25].

The dataset for this project includes only patients undergoing elective unilateral THA (not acute hip fracture or tumor surgery) who underwent surgery between 1 January 1999 and 31 December 2014. Expanding the time frame to a more recent date was impossible because the study database only contained linked data until 31 December 2015.

**Figure 1:**
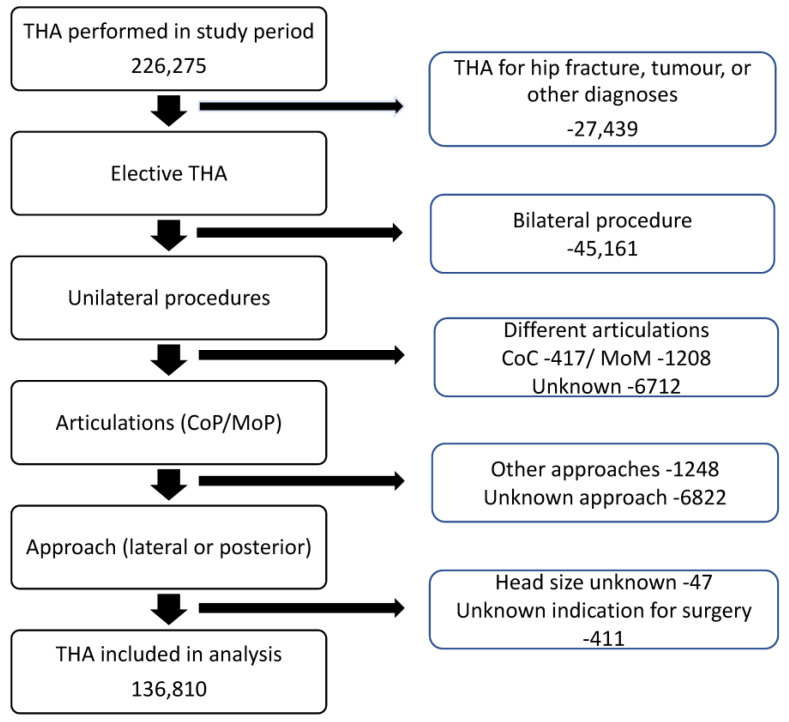
Flowchart of patient selection with inclusion and exclusion criteria (THA = total hip arthroplasty, CoC = ceramic on ceramic, MoM = metal on metal, CoP = ceramic on polyethylene, MoP = metal on polyethylene).

With the aim of describing the cumulative dislocation incidence, any potential diagnostic and procedural codes from the NPR and the SAR indicating the occurrence of this complication were identified. The ICD-10 codes used were M24.3-4, M24.4F, S73.0, T93.3 (the International Classification of Diseases), and all NFH-codes in the NOMESCO system (Nordic Medico-Statistical Committee). The diagnostic algorithm was previously used and validated by a Danish research group [26]. Patients with preoperative neurological disorders were identified using predefined codes from the NPR, and patients with spinal problems were identified using procedural and diagnostic codes within the NPR (see Supplementary Table S1). The Elixhauser comorbidity index (ECI) was calculated based on diagnoses registered within the NPR in the year preceding the index surgery. A flowchart illustrating the patient selection and the exclusion criteria for this analysis is presented in Figure 1.

Statistical analysis: The cumulative dislocation incidence and the time from the index THA to the first dislocation or to revision (for any cause or for dislocation/subluxation/instability) were estimated using the Kaplan–Meier method, giving cumulative dislocation and revision incidences at 30 and 90 days and after 1, 5, 10, and 15 years. All patients were followed up until revision, death, contralateral hip arthroplasty (for any reason), or until the end of the study period (31 December 2015), whichever came first. All observations were censored for death, revision (cup, stem, or both), or the end of follow-up. The covariates (patient- and surgery-related characteristics) from the linked database were chosen based on covariates in other peer-reviewed articles studying dislocation after elective THA [5,22,27]. The number of dislocations within 1 year of primary surgery was recorded, and the time from the first to second dislocation was estimated using the Kaplan–Meier method. Patient-related and surgery-related characteristics were compared between the cohorts of patients with a single recorded dislocation and those with multiple dislocations (>1) using Student’s *t*-test for continuous variables and the chi-squared test for categorical variables. We chose not to compare the above groups of patients with single or multiple dislocations with patients who did not have a dislocation recorded. The Kaplan–Meier method was used to analyze the time between dislocation and first revision for any cause and revisions for dislocation/subluxation/instability. For this analysis, patients with a dislocation in the first year were included, whereas all patients with a revision prior to dislocation, patients who underwent a second side surgery within a year, and patients who died before the end of a 2-year follow-up or had their surgery in 2014 were excluded. Finally, patient-related and surgery-related characteristics were compared between the cohorts of patients with dislocation within 1 year of primary surgery that were revised for any cause and those who were not revised using Student’s *t*-test for continuous variables and the chi-squared test for categorical variables. For this analysis, all patients with one or more dislocations were included.

R version 3.6.1 statistical software was used for analysis, and *p* values < 0.05 were considered statistically significant. Schoenfeld residuals were used to assess whether the proportional hazard assumption was met.

Ethical approval: Approval from the Regional Ethical Board in Gothenburg (Sweden) (271-14 and 430-15) was received on 9 April 2014 and 7 July 2015, respectively. Patients are informed about registration in the SAR at the time of their arthroplasty procedure and have the possibility to decline participation. As the patient information also mentions that register data can be used in research, no further informed consent is necessary. The study adhered to the Strengthening the Reporting of Observational Studies in Epidemiology (STROBE) guidelines and the Declaration of Helsinki [28].

## 3. Results

A total of 136,810 patients undergoing an elective unilateral THA and with a full dataset, with the exception of data missing on hospital category for 0.7% of the study population, were available for the analysis (Table 1). The median follow-up was 4.7 years (IQR 2.1,8.1).

**Figure 2:**
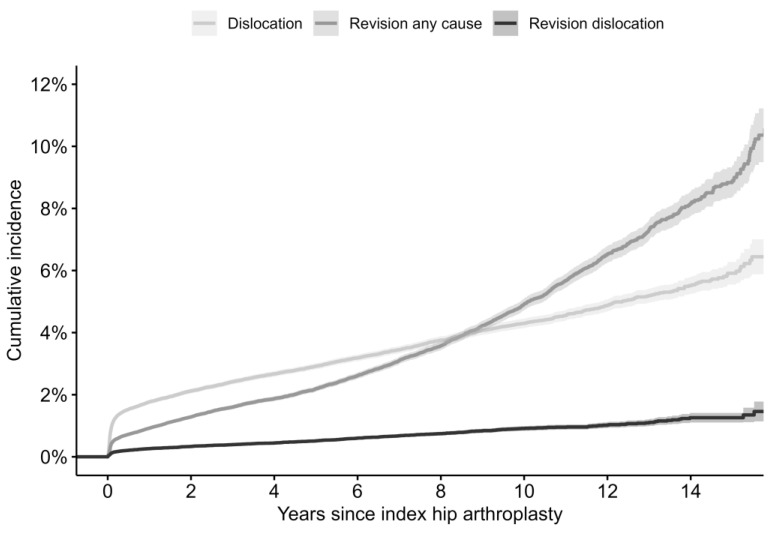
Kaplan–Meier cumulative dislocation incidence, revision for dislocation incidence, and revision for any cause incidence.

The 30-day cumulative dislocation incidence was estimated at 0.9% (0.9–1.0) (Figure 2). The revision incidence for dislocation remained throughout the study period much lower than the cumulative dislocation incidence (Figure 2). The revision incidence for any reason was lower than the dislocation incidence until 8 years post-surgery (Table 2).

**Figure 3:**
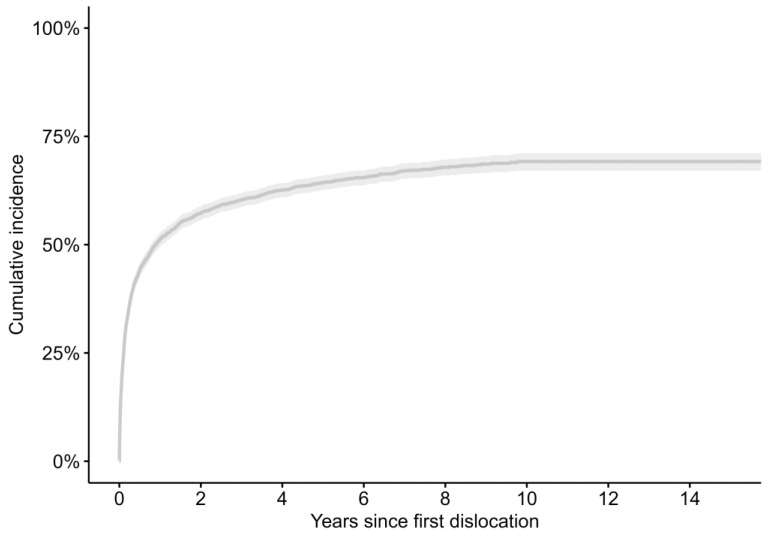
Kaplan–Meier estimate in years for time between first and second dislocation.

Using the Kaplan–Meier estimate, of the 4176 patients suffering from a dislocation within the complete study period, 22.3% (CI 21.0–23.6) suffered a further dislocation within 30 days, 36.0% (CI 34.4–37.5) within 90 days, and 51.2% (CI 49.6–52.8) within 1 year. The median time between the first and second dislocation was 10.7 months. The Kaplan–Meier estimate in years for time between the first and second dislocation is represented in Figure 3. We then focused on patients suffering a dislocation within 1 year after index surgery; 1178 patients experienced a single dislocation, whilst 1049 had multiple dislocations. Of those patients with multiple dislocations (1049), 480 patients (41%) went on to have one further dislocation, and 247 patients (21%) had a third dislocation within the first year. Patients with a THA for AVN or inflammatory arthritis, operated on in university or regional hospitals, with a known pre-existing neurological disorder or higher comorbidity as calculated using the Elixhauser comorbidity index were more common among those with multiple dislocations than among those with only one dislocation (Table 3).

**Figure 4:**
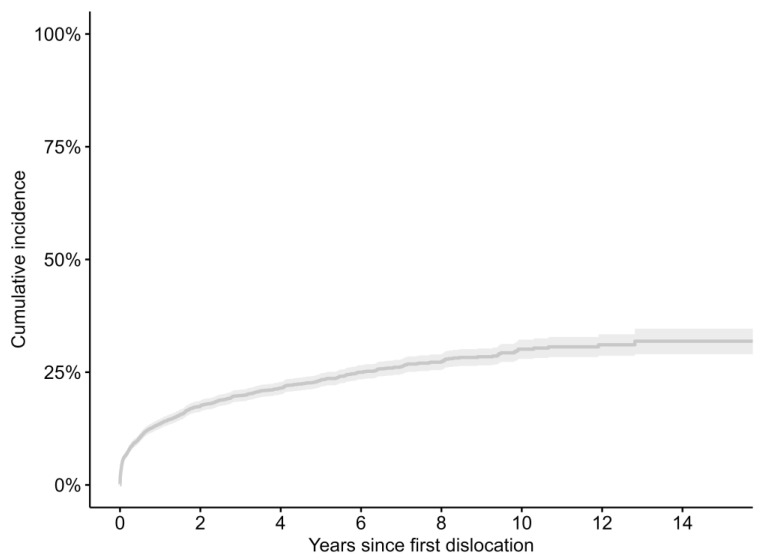
Kaplan–Meier estimate in years for time between first dislocation and revision for any cause.

The Kaplan–Meier estimate for time to revision from first dislocation to revision for any cause is represented in Figure 4. There is a difference between the Kaplan–Meier estimate for time to revision for any cause and revision for dislocation/subluxation/instability (Table 4). Patients operated on in private or university and regional hospitals were more likely to undergo revision, as were patients who had their initial surgery via a lateral approach or anything other than a fully cemented implant. Younger patients or patients suffering more than one dislocation were also more likely to have revision surgery (Table 5).

## 4. Discussion

Our findings indicate that the endpoint “revision for dislocation” clearly underestimates the dislocation burden after elective THA. The overall revision incidence for dislocation underestimates the dislocation by a factor of 1:6 at 1 year. Since dislocation as a complication of THA confers severe implications for the patient as well as the healthcare system, it is important to measure and describe dislocations and reoccurring dislocations, but also study the factors associated with suffering multiple dislocations and analyze the potential relationships with dislocation and revision surgery.

To our knowledge, this is the first study comparing the cumulative dislocation incidence and the revision incidence for dislocation and all causes following primary THA. Whilst the national cumulative rate for dislocation has previously been described by Hermansen et al., the difference in patient- and surgery-related characteristics between the patients with a single dislocation and those with multiple dislocations and between those who undergo revision and those who do not undergo revision is innovative [14].

The cumulative dislocation incidence in Sweden is lower (2.1%) than the previously published cumulative rate from a nationwide study in Denmark (3.5% at 2 years) and lower than three large cohort studies from the USA (2.3%, 2.8%, and 3.8%, respectively, at 2 years) [4,5,14,29,30]. The reasons for this could be multifactorial, such as differences in implant fixation, approach, and indication for the primary THA. The overall revision incidence for dislocation underestimates the dislocation by a factor of 1:6 at 1 year.

The number of patients having recurrent dislocations is slightly higher than previous published data from the Mayo group (44%), a study from Denmark (42%), and a study from Japan (40%), but the time frames seem to be different [4,14,31,32]. Late presentation of dislocation as a result of polyethylene wear is a concern as Sweden was late in the adoption of HXL-PE and, hence, the results of late dislocation could be elevated because of the use of the traditional PE associated with wear, catastrophic failure, and dislocation. As more than half of the patients in Sweden will encounter one or more further dislocations within 1 year, it is important to understand which prospectively recorded, patient- and surgery-related are relevant. BMI data were not analyzed as the BMI has only been routinely recorded since 2008 [33]. In contrast to the study from the Mayo clinic and similar to the outcome of the study from Japan, there was no difference in sex between the group of those who suffered a single dislocation versus the group of multiple dislocations; there was, however, an association between the risk of recurrence of dislocation and the diagnosis at the time of primary THA, with more dislocations in patients who had a THA for inflammatory arthritis or avascular necrosis [31,32]. The association between surgery at university hospitals and reoccurring dislocations is likely to be multifactorial, but the complexity of the cases might be an important factor.

Revision following dislocation following elective primary THA has been published to be between 13.5% and 46% [4,30,31,34]. Revision surgery following dislocation occurs later and less so than in a previous published study from the USA as, at 2 years, only 17.5% of the patients with a dislocation will have had revision surgery recorded versus 46% [4]. However, a more recent analysis using Medicare data from the USA found that only 13.5% of the patients suffering a dislocation undergo revision surgery [30]. Revision for dislocation remains one of the top indications of revision procedures in many register reports, although there are some reports stating that the issue is becoming less of a problem, and perhaps the larger head sizes and the use of DM-THC may have contributed to this improvement [30].

This study has several inherent limitations. As with any cohort and register study, there is a potential for confounding by indication. A study of an observational nature, based on the available data, always carries a degree of selection bias. However, this is unlikely but inevitable as some patients with a known dislocation might not be offered revision surgery for many reasons or following discussion between the patient and surgical team as part of the shared decision-making process [35,36,37]. Unknown or unrecorded confounders include implant positioning or spinopelvic orientation. There is a lack of radiological assessment of the position of the implants and correction of offset, and malpositioning is not recorded in SAR as a reason for revision. Previous studies have described safe zones, and the influence of the spinopelvic relation (position and mobility) is a recognized risk factor for dislocation [38]. It is also a limitation that the results cannot be generalizable to a population operated on through an anterior approach. It is impossible to analyze the dislocation incidence in the direct anterior approach or to depict the association of the anterior approach with dislocation, as the anterior approach has been very rarely used as an approach in Sweden. Further on, the study group was restricted to unilateral hips only and so a large proportion of the population with bilateral THAs is excluded [39]. Previous studies have identified that after 10 years, around 25% of patients will have undergone a contralateral THA [39]. We also used only preoperatively documented comorbidity, neurological disorders, and spinal problems. Those patients who subsequently postoperatively developed the above medical concerns could well be misidentified. The strengths of this study are the nationwide prospective collection of data, the high coverage and completeness, and the linkage of the data. A final limitation is the limited number of head sizes of 36 mm and dual mobility cups within the studied cohort as Swedish surgeons have traditionally used 32 mm heads for their primary THAs.

## 5. Conclusions

The dislocation incidence after elective THA was expectedly considerably higher than the revision incidence for dislocation; hence, this analysis would caution against using the revision incidence for dislocations as a proxy for the dislocation incidence. Following a first-time dislocation, 48.8% will not have a further dislocation within a year, and most patients will not undergo a revision for dislocation. Further studies investigating the differences between single and multiple dislocators and the criteria by which patients are offered revision surgery following dislocation are urgently needed.

## Figures and Tables

**Table 1:** Characteristics of the study population (OA = osteoarthritis, cTHA = conventional total hip arthroplasty, THA-DMC = total hip arthroplasty with dual mobility cup, SD = standard deviation).

		Absolute Number	%
Study group		136,810	100
Indication for primary surgery	Primary OA	122,751	89.7
	Secondary OA, unspecified	5139	3.8
	Sequelae from childhood hip disorders	3205	2.3
	Inflammatory joint disease	2832	2.1
	Avascular necrosis femoral head (primary)	2877	2.1
Sex	Female	77,644	57.8
	Male	59,166	43.2
Hospital category	University or regional hospitals	12,773	9.4
	County hospitals	46,021	33.9
	Rural hospitals	55,489	40.9
	Private hospitals	21,505	15.8
Fixation	Fully cemented	102,659	75.0
	Hybrid	4986	3.6
	Reverse hybrid	12,732	9.3
	Uncemented	16,433	12.0
Approach	Posterior	77,013	56.3
	Lateral	59,797	43.7
Bearing type and size	cTHA < 32 mm	89,608	65.5
	cTHA 32 mm	40,930	29.9
	cTHA > 32 mm	4670	3.4
	THA-DMC	1602	1.2
Year of surgery	1999–2000	6428	4.7
	2001–2002	14,353	10.5
	2003–2004	16,507	12.1
	2005–2006	18,116	13.2
	2007–2008	18,344	13.4
	2009–2010	20,843	15.2
	2011–2012	21,024	15.4
	2013–2014	21,205	15.5
Preoperative diagnosis of a neurological disorder	Yes	3013	2.2
	No	133,797	97.8
Preoperative diagnosis of spinal disease	Yes	11,573	8.5
	No	125,237	91.5
Elixhauser comorbidity index (mean) (SD)		0.58 (0.96)	
Elixhauser comorbidity category	0	87,236	63.8
	1	29,804	21.8
	2	12,727	9.3
	3	4739	3.5
	4+	2304	1.7
Age (mean) SD		67.9 (10.7)	
Age category	<55	14,393	10.5
	55–69	58,035	42.4
	70–84	59,038	43.2
	85+	5344	3.9

**Table 2:** Cumulative dislocation, revision for dislocation, and revision for any cause incidences with 95% confidence intervals at different time points following elective THA (1—Kaplan–Meier estimates).

	Cumulative Dislocation Incidence (%)	Cumulative Revision for Dislocation Incidence (%)	Cumulative Revision for Any Reason Incidence (%)
30 days	0.9 (0.9–1.0)	0.1 (0.1–0.1)	0.4 (0.4–0.4)
90 days	1.3 (1.3–1.4)	0.2 (0.1–0.2)	0.6 (0.6–0.6)
1 year	1.8 (1.7–1.8)	0.3 (0.2–0.3)	0.9 (0.9–1.0)
2 years	2.1 (2.0–2.2)	0.3 (0.3–0.4)	1.3 (1.2–1.3)
3 years	2.4 (2.3–2.5)	0.4 (0.4–0.4)	1.6 (1.5–1.7)
4 years	2.7 (2.6–2.8)	0.4 (0.4–0.5)	1.9 (1.8–1.9)
5 years	2.9 (2.8–3.0)	0.5 (0.5–0.6)	2.2 (2.1–2.3)
10 years	4.3 (4.1–4.4)	0.9 (0.8–1.0)	4.9 (4.7–5.1)
15 years	5.9 (5.5–6.3)	1.3 (1.1–1.4)	8.9 (8.4–9.4)

**Table 3:** Difference in patient- and surgery-related characteristics in patients with single or multiple dislocations within 1 year of primary THA.

		Single Dislocation	Multiple Dislocations	*p*-Value
Study group		1178	1049	
Indication for primary surgery (%)	Primary OA	1017 (86.3)	867 (82.7)	0.035
	Secondary OA, unspecified	64 (5.4)	56 (5.3)	
	Sequelae from childhood hip disorders	20 (1.7)	18 (1.7)	
	Inflammatory joint disease	31 (2.6)	43 (4.1)	
	Avascular necrosis femoral head (primary)	46 (3.9)	65 (6.2)	
Sex (%)	Female	606 (51.4)	538 (51.3)	0.975
	Male	572 (48.6)	511 (48.7)	
Hospital category (%)	University or regional hospitals	87 (7.5)	119 (11.4)	0.001
	County hospitals	446 (38.3)	414 (39.5)	
	Rural hospitals	455 (39.0)	396 (37.8)	
	Private hospitals	178 (15.3)	118 (11.3)	
Fixation (%)	Fully cemented	948 (80.5)	851 (81.1)	0.514
	Hybrid	34 (2.9)	33 (3.1)	
	Reverse hybrid	79 (6.7)	55 (5.2)	
	Uncemented	117 (9.9)	110 (10.5)	
Approach (%)	Posterior	848 (72.0)	752 (71.7)	0.913
	Lateral	330 (28.0)	297 (28.3)	
Bearing type and size (%)	cTHA < 32 mm	838 (71.1)	764 (72.8)	0.808
	cTHA 32 mm	305 (25.9)	256 (24.4)	
	cTHA > 32 mm	33 (2.8)	28 (2.7)	
	THA-DMC	2 (0.2)	1 (0.1)	
Year of surgery (%)	1999–2000	69 (5.9)	39 (3.7)	0.115
	2001–2002	140 (11.9)	126 (12.0)	
	2003–2004	160 (13.6)	150 (14.3)	
	2005–2006	153 (13.0)	166 (15.8)	
	2007–2008	163 (13.8)	150 (14.3)	
	2009–2010	170 (14.4)	159 (15.2)	
	2011–2012	168 (14.3)	123 (11.7)	
	2013–2014	155 (13.2)	136 (13.0)	
Preoperative diagnosis of a neurological disorder (%)	Yes	45 (3.8)	60 (5.7)	0.044
	No	1133 (96.2)	989 (94.3)	
Preoperative diagnosis of spinal disease (%)	Yes	155 (13.2)	139 (13.3)	0.998
	No	1023 (86.8)	910 (86.7)	
Elixhauser comorbidity index (mean) (SD)		0.75 (1.10)	0.95 (1.17)	<0.001
Elixhauser category	0	667 (56.6)	485 (46.2)	0.524
	1	292 (24.8)	301 (28.7)	
	2	129 (11.0)	162 (15.4)	
	3	51 (4.3)	57 (5.4)	
	4+	39 (3.3)	44 (4.2)	
Age (mean) (SD)		70.40 (10.28)	71.28 (10.05)	0.044
Age category (%)	<55	75 (6.4)	64 (6.1)	0.495
	55–69	445 (37.8)	367 (35.0)	
	70–84	585 (49.7)	544 (51.9)	
	85+	73 (6.2)	74 (7.1)	

**Table 4:** Kaplan–Meier estimates for time from dislocation to revision for dislocation/subluxation/instability and revision for any cause with confidence intervals (CI) (1—Kaplan–Meier estimates).

	Kaplan–Meier Estimates for Time between Dislocation and Revision for Dislocation (%) (95% CI)	Kaplan–Meier Estimates for Time between Dislocation and Revision for Any Cause (%) (95% CI)
30 days	4.2 (3.6–4.8)	5.8 (5.1–6.5)
90 days	6.1 (5.3–6.8)	8.2 (7.3–9)
6 months	8.2 (7.3–9)	10.5 (9.5–11.4)
1 year	10.9 (9.9–11.9)	13.6 (12.5–14.6)
2 years	14.5 (13.3–15.6)	17.5 (16.2–18.7)
3 years	16.4 (15.2–17.7)	19.8 (18.5–21.1)
4 years	17.8 (16.5–19.1)	21.5 (20.1–22.8)
5 years	19.4 (18–20.8)	23.3 (21.8–24.8)
10 years	24.3 (22.4–26.2)	30.1 (27.9–32.2)
15 years	24.6 (22.6–26.5)	31.9 (29–34.7)

**Table 5:** Difference in patient- and surgery-related characteristics in patients revised and non-revised following dislocation within 1 year of primary THA.

		Not Revised within a Year after First Dislocation	Revised within a Year after First Dislocation	*p*-Value
Study group		1677	296	
Indication for primary surgery (%)	Primary OA	1427 (85.1)	243 (82.1)	0.171
	Secondary OA, unspecified	95 (5.7)	15 (5.1)	
	Sequelae from childhood hip disorders	25 (1.5)	10 (3.4)	
	Inflammatory joint disease	56 (3.3)	12 (4.1)	
	Avascular necrosis femoral head (primary)	74 (4.4)	16 (5.4)	
Sex (%)	Female	871 (51.9)	152 (51.4)	0.902
	Male	806 (48.1)	144 (48.6)	
Hospital category (%)	University or regional hospitals	141 (8.4)	39 (13.2)	0.004
	County hospitals	672 (40.3)	99 (33.6)	
	Rural hospitals	658 (39.4)	109 (36.9)	
	Private hospitals	198 (11.9)	48 (16.3)	
Fixation (%)	Fully cemented	1415 (84.4)	212 (71.6)	<0.001
	Hybrid	49 (2.9)	11 (3.7)	
	Reverse hybrid	86 (5.1)	31 (10.5)	
	Uncemented	127 (7.6)	42 (14.2)	
Approach (%)	Posterior	1256 (74.9)	171 (57.8)	<0.001
	Lateral	421 (25.1)	125 (42.2)	
Bearing type and size (%)	cTHA < 32 mm	1284 (76.6)	214 (72.3)	0.344
	cTHA 32 mm	351 (20.9)	74 (25.0)	
	cTHA > 32 mm	40 (2.4)	7 (2.4)	
	THA-DMC	2 (0.1)	1 (0.3)	
Year of surgery (%)	1999–2000	89 (5.3)	11 (3.7)	0.010
	2001–2002	224 (13.4)	27 (9.1)	
	2003–2004	255 (15.2)	43 (14.5)	
	2005–2006	260 (15.5)	45 (15.2)	
	2007–2008	242 (14.4)	59 (19.9)	
	2009–2010	263 (15.7)	43 (14.5)	
	2011–2012	239 (14.3)	36 (12.2)	
	2013–2014	105 (6.3)	32 (10.8)	
Preoperative diagnosis of a neurological disorder (%)	Yes	71 (4.2)	19 (6.4)	0.131
	No	1606 (95.8)	277 (93.4)	
Preoperative diagnosis of spinal disease (%)	Yes	198 (11.8)	40 (13.5)	0.463
	No	1479 (88.2)	256 (86.5)	
Elixhauser comorbidity index (mean) (SD)		0.80 (1.11)	0.87 (1.11)	0.320
Elixhauser category	0	888 (53.0)	142 (48.0)	0.524
	1	450 (26.8)	89 (30.1)	
	2	209 (12.5)	40 (13.5)	
	3	72 (4.3)	16 (5.4)	
	4+	58 (3.5)	9 (3.0)	
Age (mean) (SD)		70.94 (10.03)	69.35 (10.47)	0.013
Age category (%)	<55	92 (5.5)	27 (9.1)	0.050
	55–69	619 (36.9)	115 (38.9)	
	70–84	856 (51.0)	140 (47.3)	
	85+	110 (6.6)	14 (4.7)	
Number of dislocations in the first year (mean (SD))		1.78 (1.27)	3.81 (2.63)	<0.001
Number of patients with multiple dislocations in the first year (%)		698 (41.6)	237 (80.1)	<0.001

## Data Availability

Data are available on request due to restrictions.

## Supplementary Materials

The following supporting information can be downloaded at: https://www.mdpi.com/article/10.3390/jcm13020598/s1, Table S1: Preoperative neurological disorders were identified using predefined codes from the National Patient Regsiter(NPR), and patients with spinal problems were identified using procedural and diagnostic codes within the NPR. (NA = Not applicable).
