# Supplementary material for: The True Dislocation Incidence following Elective Total Hip Replacement in Sweden: How Does It Relate to the Revision Rate?
Source: J Clin Med. 2024 Jan 20;13(2):598. doi: 10.3390/jcm13020598 (PMC10816596; doi:10.3390/jcm13020598)
Supplement: Supplementary file 1 [file jcm-13-00598-s001.zip › jcm-2800302-supplementary.pdf]

Supplementary Materials

SUPPLEMENTARY TABLE S1				
	Variable name	Diagnoses	Interventions	Comment
**Dislocations**	Dislocation	M243, M244, M244F, S730, T933	NFH00-NFH30, NFH40, NFH42, NFH70-NFH99	NA
**Preoperative diagnosis of spinal problems or spinal surgery**	Preoperative diagnosis of spinal problems	ABC07, ABC16, ABC26, ABC36, ABC40, ABC56, ABC66, ABC99, NAB94, NAC94, NAG39, NAG49, NAG59, NAG69, NAG79, NAG89, NAG99	NFH00, NFH01, NFH02, NFH03, NFH04, NFH05, NFH06, NFH07, NFH08, NFH09, NFH10, NFH11, NFH12, NFH13, NFH14, NFH15, NFH16, NFH17, NFH18, NFH19, NFH20, NFH21, NFH22, NFH23, NFH24, NFH25, NFH26, NFH27, NFH28, NFH29, NFH30, NFH40, NFH41, NFH42, NFH70, NFH71, NFH72, NFH73NFH74, NFH75, NFH76, NFH77, NFH78, NFH79, NFH80, NFH81, NFH82, NFH83, NFH84, NFH85, NFH86, NFH87, NFH88, NFH89, NFH90, NFH91, NFH92, NFH93, NFH94, NFH95, NFH96, NFH97, NFH98, NFH99	Yes, if a primary or secondary diagnosis at any time prior to index operation
**Preoperative diagnosis of neurological disorder**	Preoperative diagnosis of neurological disorder _neuro	G10, G11, G12, G20, G21, G22, G30, G31, G35, G70, G71, G72, G73, G40, F039, F019, F001, F002, F009, F012, F011, F107A, F107W, F103, F023, F028, F000, F020, F018, F010, F021, F022,	NA	Yes, if a primary or secondary diagnosis at any time prior to index operation
